# Attachment style, anxiety coping, and personality-styles in withdrawn alcohol addicted inpatients

**DOI:** 10.1186/1747-597X-8-1

**Published:** 2013-01-10

**Authors:** Dirk Wedekind, Borwin Bandelow, Soren Heitmann, Ursula Havemann-Reinecke, Kirsten R Engel, Gerald Huether

**Affiliations:** 1 Department of Psychiatry and Psychotherapy, University of Göttingen, von Siebold Strasse 5, 37075, Goettingen, Germany

**Keywords:** Alcohol addiction, Attachment, Personality style, Anxiety

## Abstract

**Background:**

Insecure early attachment experiences have been reported to play an important role in the manifestation in alcoholism. The purpose of this study was to investigate the relationship of attachment styles with anxiety, anxiety coping and dysfunctional personality styles, as well as with the prevalence of personality disorders, and adverse life-events in adolescence.

**Methods:**

59 inpatient alcohol addicted male (n=43) and female (n=16) patients were characterized by an attachment style scale (Relationships-style-questionnaire-RSQ) and completed a questionnaire battery comprising the State-Trait-Anxiety-Inventory (STAI), the Anxiety-Coping-Inventory (ABI), Temperament-and-character-inventory (TCI), Personality-system-interaction-inventory (PSI), and gave information on sociodemography, alcohol history, and adolescent adverse events. A structured interview (SKID-II) was performed to diagnose personality disorders.

**Results:**

Only 33% of subjects had a secure attachment style. Insecure attachment was associated with significantly higher trait-anxiety, higher cognitive avoidance to control anxiety, and higher values on most personality style dimensions directed to the pathological pole.

**Conclusions:**

Despite the limitation due to a small sample size, the results of this study show that the consideration of attachment styles is of significance in the diagnosis and therapy of alcohol addiction. Attachment may characterize different styles to control emotional aspects, anxiety cues and interpersonal relationships in individuals suffering from alcohol addiction.

## Introduction

Alcohol addiction is a complex disorder with a high overall prevalence [1-3] and a high impact on health related and socioeconomic aspects. Several factors are involved in the development of alcohol addiction including genetic predispositions as well as neuro- and socio-developmental phenomena.

Attachment research has repeatedly shown the influence of early social interactions on developing inner working models and object representations that substantially impact on bonding behaviour, subject-environment-interactions and psychopathology in later life [4-7]. On the other hand attachment may also be determined by genetic and biological aspects to a higher extent than previously thought [8-14]. According to Bowlby’s conceptual description [15], interactions of genetic, neurobiological, and developmental factors contribute to the establishment of neuronal networks involved in the regulation of stress resilience, anxiety sensitivity and personality development. All three variables must therefore be supposed to contribute to the development of certain psychopathologies or even psychiatric disorders under certain circumstances (e.g. [16,17]).

Ainsworth et al. [18], first described different behavioural patterns, so-called “attachment styles” which have subsequently been implemented in various reliable and valid rating scales for the differentiation between individual attachment styles (secure, insecure avoidant, insecure anxious, insecure ambivalent and disorganised). Several reports indicate a higher prevalence of insecure compared to secure attachment styles in alcohol addiction [19-24] and other psychiatric disorders. Subjects with insecure attachment styles have a reduced capability of experiencing reliability, trust, and safety through relationships. For the infant, secure attachment is a crucial factor for its emotional self-regulation. Developmental psychology could demonstrate that in early childhood the perception of potentially threatening objects and situation is basically dependent on the emotional reaction of the parent. Constant experience of availability and reliability of such a stress-reducing and emotionality regulating object is likely to be associated with a secure attachment style in later life. If this available and reliable attachment is experienced insufficiently, the individuals coping of stress or anxiety is therefore less effective and this may activate epigenetic mechanisms promoting onset of psychiatric disorders later on. Additionally, insecure attachment styles may go along with the development of distinct potentially dysfunctional behaviours in social interactions promoting the development of dysfunctional personality styles or personality disorders (e.g. [24]). This dysfunctionality is related to a higher incidence of addictive disorders [1,25-27]. Nevertheless, an insecure attachment style is not necessarily pathological. Insecure attachment may also lead to “qualified justification” [28] as an essential contributing factor of the functioning of societies. However, neglected, violated, or traumatised insecurely attached individuals are very likely to apply educational styles which contribute to insecure attachment in their offspring [29]. Such children develop behavioural and emotional problems more frequently in childhood and adolescence. The same is true for addiction which is regarded as an attachment disorder by some authors (e.g. [30]). Potentially harmful drinking patterns have been associated with insecure attachment and dysfunctional capacities to regulate emotions [31]. Recently, De Rick and co-workers have suggested a correspondence between personality disorder traits, anxiety and depression in alcohol addiction [20].

In order to further clarify this relationship, the present study investigates how secure versus insecure attachment styles in inpatient alcohol addicted subjects correspond with trait anxiety symptoms and anxiety coping style as well as on personality disorders and styles and distinct dimensions of personality characterised by temperament and character-related features according to Cloninger’s tridimensional personality theory [32]. Cloninger differentiated more biologically determined temperament variables (namely novelty seeking, harm-avoidance, reward-dependence, and perseverance) which have associations to certain monoamine transmitter systems from character dimensions. These are self-directedness, cooperativeness, and self-transcendence. Self-directedness is a character-dimension which represents the individual ability to control emotionality by self-initiated behaviour; cooperativeness is described as an ability to control emotional states by reliable social interaction. Both dimensions suggest a potentially close relationship to features of the attachment style.

We hypothesized that insecurely attached alcohol addicts will have a higher prevalence of basic anxiety symptoms and dysfunctional anxiety coping styles as well as a higher occurrence of dysfunctional personality styles and less beneficial expression of temperament and character dimensions according to Cloninger’s theory. Furthermore we investigated whether the prevalence of adverse, potentially traumatizing life-events had a higher prevalence in insecurely attached alcohol dependent patients.

## Methods and subjects

The study was approved by the local ethics committee of the medical faculty of the University of Goettingen.

### Study sample

59 male (n=43) and female (n=16) subjects undergoing qualified inpatient detoxification treatment volunteered for the study. Patients were consecutively asked to participate over the study period between June 2004 and May 2005. No remuneration or other incentives for participants were provided.

Subjects were between 24 and 70 years old (mean 46.1 ± 10.0 years (males 45.3 ± 10.0; females 48.2 ± 9.9). Patients had to meet diagnostic criteria of alcohol addiction according to ICD-10 [33]. Patients were interviewed 3–10 days after acute withdrawal symptoms had ceased and after having given informed written consent. Subjects younger than 18 or older than 70 years of age were not included. Neither included were addicts with polyvalent substance addiction (part from nicotine), schizophrenia or other psychotic disorder, severe affective disorder or relevant organic brain disorder, or incapacitating somatic disorders. Data was collected at two sites specified for alcohol addiction treatment. Examinations were done by two experienced clinicians.

### Interview procedure

Examinations during this study consisted of self- and clinician rated scales and interviews. The entire examination took approximately 90 minutes.

The clinician performed a structured interview on personality disorders according to DSM-IV [34] (SKID II) and completed a questionnaire raising data on number of previous alcohol detoxification treatments, duration of alcohol addiction, education (level of education and job qualification was measured on a 0–4 nominal-scale) and admission to the clinic.

Participants completed a sample of questionnaires listed below. The questions of the FPI-R [35] openness-scale were integrated in the sample.

#### Relationships-style-questionnaire – RSQ

The RSQ [36] is a valid instrument for the self-reported measurement of adult attachment styles. It allows discriminating between 1) secure, 2) insecure ambivalent, 3) insecure dismissive, 4) insecure avoidant.

#### State-Trait-Anxiety-Inventory – STAI

The STAI [37] is an established and valid self-rating instrument to measure momentary (STAI-1) and chronic anxiety symptoms (STAI-2) in adult subjects. Both are to be rated on separate scales so that differential statements can be drawn. Values are an the range between a minimum of 20 and a maximum of 80 points for each scale.

#### Anxiety-coping-inventory – ABI

The ABI [38] is a validated self-rating scale according to Krohne’s theory of anxiety coping. Krohne’s theory differentiates anxiety “repressers” who cognitively avoid anxiety related stimuli and “sensitizers” who react to anxiety related stimuli with more vigilance to gain more information on the stimulus in order to reduce insecurity. The ABI (German version “Angstbewaeltigungs-Inventar”) is a validated scale to differentiate between anxiety repressers and sensitizers.

#### Temperament and character inventory – TCI

Cloninger’s tridimensional personality theory is based on the existence of experience dependent character traits and more genetically and biologically determined temperament features. The TCI [32] is a reliable and valid self-rating instrument to measure Cloninger’ s personality dimensions. Cloninger’ s theory distinguishes distinct temperament dimensions such as novelty seeking, harm-avoidance, reward-dependence, perseverance and character dimensions. Self-directedness represents the individual’ s experience of being able to influence his environment by voluntary behaviour and emotions, cooperativeness represents the individuals experiencing of being effective part of a social group, and self-transcendence represents the individual experience of being part of universe. Raw data can be transferred to values related to a normative sample.

#### Personality-system-interaction-inventory – PSI

The PSI-theory by Kuhl focuses on a timely dimensional understanding of functional personality features up to dysfunctional traits interfering with functioning. Dimensions of the PSI [39] are arranged in dimensional poles (e.g. loyal-dependent or critical-negativistic) characterizing extremes of traits. Raw values can be transferred according to normative data.

#### Traumatic experience in adolescence questionnaire

The traumatic experience questionnaire [40] is a 203 item instrument to gain information on potentially traumatic events in adolescence. Items are asked on an ordinal or nominal scale level asking whether certain events (like prevalence of psychiatric disorders in parents, physical abuse in adolescence) occurred and how severely they were perceived. Information about perception of parental care is obtained.

### Statistical evaluation

Differences between the two groups having secure and insecure attachment were calculated with regard to age (t-test) and gender (Fisher’s exact-test).

Correlations between attachment style and anxiety-coping style were calculated by t-test and group differences between attachment styles and TCI and PSI-scores were computed by t-test. In order to keep the α-error low, a correction according to the Bonferroni-Holm method was applied for the multiple calculations on the PSI- (level of significance adapted to 0.00426) and the TCT-scale (level of significance adapted to 0.0073). For all other calculations the threshold of significance was set at .05.

Correlations between attachment style and prevalence of personality disorders were calculated by Chi^2^ test. Differences on the applied scales were computed for differences between secure and insecure attachment, insecure attachment was not subdivided in distinct insecure styles or by sex due to low n.

## Results

The prevalence of the secure attachment style in the sample was 33% and 67% for the insecure attachment style. Differences in the distribution between sexes were not significant. Secure attachment was found in 35% of males and 31% of females. Insecure attachment styles were accordingly found in 65% of males and 69% of females and could be distinguished into 24% dismissive (males 21%, females 31%), 24% ambivalent (males 23%, females 25%), and 19% avoidant (males 21%, females 13%).

The mean age of the sample was 46.1 ± 10.0 years (males 45.3 ± 10.0; females 48.2 ± 9.9; (T-test: df= 57; t= −0.99; p= .33); sex difference not significant; range 24–70). Average duration of alcohol addiction was 13.3 ± 11.5 years for the entire sample, 13.1 ± 11.3 for males and 13.8 ± 12.0 for females (T-Test: df=57; t=−0.21; p=.84). Subjects had an average of 3.2 ± 2.1 inpatient detoxification treatments previously (males 4.1 ± 2.7, females 3.1 ± 1.5 (T-Test: df=57; t=1.4; p=.17)). Both differences were not significant.

The average onset of alcohol addiction in secure attachment (SAS) was not later than in insecure attachment style (IAS) and there were about the same number of previous inpatient alcohol-related treatments in secure attachment style subjects. Personality disorders as diagnosed by SKID-II were found in 8 IAS and in 1 SAS subject, however, this difference was not significant. In the SAS group on subject had symptoms of paranoid personality disorder, in the IAS group 6 of 8 subjects had more than one personality disorder.

Scores on the implied openness scale of the FPI-R which were used as a validity scale for the entire battery altogether had high scores which were comparable between SAS and IAS (20.7 ± 5.1 vs. 20.3 ± 3.8; T-test: df= 57; t=−0.34; p=.74). The level of education was similar in SAS and IAS subjects (2.56 ± .61 vs. 2.49 ± .65; (T-test: df=57; t=−0.40; p=.69). Job qualification revealed a significantly higher qualification of IAS compared to SAS patients (1.21 ± .17 vs. .81 ± .21; (T-test: df=57; t=7.89; p<.001).

Anxiety levels as revealed from the STAI showed scores in trait- and state anxiety in the entire group (see Tables 1 and 2) as could be expected from moderately anxious individuals. Alcohol addicted SAS subjects had comparable levels on state anxiety compared to IAS, trait anxiety however, was significantly higher in IAS subjects compared to SAS (Fisher’s exact-test: p=.005).

**Table 1:** Distribution of attachment styles in whole sample – subdivision by sex

	**Secure attachment**	**Insecure ambivalent**	**Insecure dismissive**	**Insecure avoidant**
Females (n=16)	31% (n=5)	31% (n=5)	25% (n=4)	13% (n=2)
Males (n=43)	35% (n=15)	21% (n=9)	23% (n=10)	21% (n=9)
**Total sample (n=59)**	**33% (n=20)**	**24% (n=14)**	**24% (n=14)**	**19% (n=11)**

**Table 2:** Personality disorders, personality styles, TCI, PSI, STAI, ABI scores and prevalence of adverse events in secure and insecure attachment styles

	**Insecure attachment style n =39**	**Secure attachment style n = 20**	**df**	**Significance**
Duration of addiction (years)	13.6 ± 11.9	12.7 ± 11.2	57	t= 0.28; p= .78
Qualified Detoxifications	4.2 ± 7.1	3.3 ± 3.7	57	t= 0.53; p= .60
Personality disorders**	8 (19%)	1 (5%)	1	p=.148
STAI-1-State anxiety	41.3 ± 4.9	42.2 ± 10.8	57	t =−0.44; p= .66
STAI-2-Trait anxiety	47.2 ± 4.9	42.6 ± 7.1	57	t =2.92; p=.005
ABI-V (sensitizers) PR	74.8 ± 26.9	76.3 ± 28.3	57	t =−0.20 ; p= .84
ABI-K (repressers) PR	43.8 ± 15.5	60.8 ± 18.7	57	t=−3.72 ; p=.001
PSI scale PR ± SD				
1 (antisocial)	56.0 ± 13.9	44.6 ± 14.0	57	t=2.98; p=.004
2 (paranoid)	73.5 ± 8.8	55.3 ± 5.9	57	t=8.32; p<.001
3 (schizoid)	74.1 ± 8.6	49.2 ± 11.2	57	t=9.48, p<.001
4 (insecure)	66.3 ± 9.0	42.9 ± 8.7	57	t=9.56; p<.001
5 (anancastic)	71.8 ± 9.8	71.4 ± 10.7	57	t=0.14; p= .89
6 (schizotypic)	54.1 ± 8.7	45.1 ± 11.6	57	t=3.35; p< .001
7 (rhapsodic)	42.7 ± 9.6	59.3 ± 10.1	57	t=−6.18; p<.001
8 (narcissic)	38.4 ± 8.7	26.6 ± 12.0	57	t=4.32; p<.001
9 (negativistic)	76.6 ± 9.9	57.9 ± 9.0	57	t=7.08; p<.001
10 (dependent)	72.3 ± 8.2	49.1 ± 10.8	57	t=9.22; p<.001
11 (borderline)	69.1 ± 7.8	49.1 ± 9.0	57	t=8.85; p<.001
12 (histrionic)	40.2 ± 9.6	59.3 ± 8.5	57	t= −7.51; p<.001
13 (depressed)	73.8 ± 8.9	52.5 ± 8.4	57	t=8.87; p<.001
14 (self-sacrificing)	80.4 ± 8.8	73.3 ± 9.8	57	t=2.82; p= .007
TCI-scale PR ± SD Self-directedness	23.5 ± 9.0	38.3 ± 11.6	58	t=−5.41; p<.001
Cooperativeness	34.4 ± 21.4	42.9 ± 18.6	58	t=−1.51; p= .14
Self-transcendence	45.6 ± 13.8	41.2 ± 20.6	58	t=0.98; p=.33
Novelty-seeking	46.6 ± 7.7	58.3 ± 6.9	58	t=−5.72; p<.001
Harm-avoidance	73.4 ± 9.9	48.2 ± 8.5	58	t=9.69; p<.001
Reward-dependence	48.7 ± 12.0	45.3 ± 14.4	58	t=0.96; p= .34
Perseverance	55.3 ± 10.5	48.0 ± 14.0	58	t=2.25; p= .028

*PR* = percent-rank. *SD*= standard deviation.

For variables from personality-scale Fisher’ s exact test was used. P-values are exact p-values.

all: T-test except **: Fisher’s exact-test used.

On the anxiety coping style according to ABI scores (see Table 2) IAS turned out to be comparably over-average sensitizers than SAS but they were significantly lower in cognitive avoidance / repression compare to the SAS group (see Table 2).

On the TCI (see Table 2) below-average scores (values below the 50% rank of the reference sample of this scale) were found for self-directedness which was significantly lower expressed in IAS compared to SAS. On the temperament scales SAS subjects scored significantly higher on novelty seeking than IAS subjects but significantly lower on harm-avoidance also revealing over-average harm-avoidance in IAS. Cooperativeness-scores were below average and for self-transcendence for both groups, however without any significant difference. Reward-dependence was comparable for both attachment styles as well as perseverance but no group differences were evident after Bonferroni-Holm correction.

Results from the PSI-scores detected interesting features matching our hypothesis. Whereas SAS subjects were about the average of normative distribution on all items, IAS-alcohol addicts where more directed to the pathological poles for most items (see Table 2). Compared to SAS subjects IAS were significantly more paranoid, schizoid, insecure, schizotypic, negativistic, dependent, borderline and depressive. SAS alcohol addicts, on the other hand, were significantly more histrionic and rhapsodic compared to IAS. No group differences were evident with regard to antisocial, anancastic, rhapsodic, narcissic, and unselfish features (see Table 2).

Adverse and potentially traumatic events in childhood and adolescence (see Table 3) showed more subjects with a prevalence of marital problems in IAS alcoholics. Childhood domestic violence and psychiatric disorder of one or both parents were similarly frequent in both groups. Sexual molestation in adolescence was found in IAS and in SAS subjects, results were not significant.

**Table 3:** Adverse events in adolescence in secure and insecure attachment (1–5:fisher’s exact-test; 6–7: T-test)

	**Insecure attachment style n = 39**	**Secure attachment style n = 20**	**df**	**Significance**
1)Parents’ marital problems	56% (n=22)	15% (n=3)	1	p= .025
2)Parents’ alcoholism	36% (n=14)	29% (n=4)	1	p= .25
3)Parents’domestic violence	21% (n=8)	25% (n=5)	1	p= .75
4)Parents’psychiatric diagnosis	15.4% (n=6)	10% (n=2)	1	p= .70
5)Sexual molestation	13% (n=5)	5% (n=1)	1	p= .65
6)Father’s care (0–4)	2.4 ± 1.4	3.0 ± 1.6	57	t= −1.48; p= .14
7)Mother’s care (0–4)	2.5 ± 1.5	2.9 ± 1.6	57	t=−0.95 p= .35

Perception of parental care in childhood and adolescence was described as fairly good (on a 0–4 scale) and did not differ between groups.

## Discussion

Recent attachment research has indicated that besides the high relevance of early childhood experiences also biological variables account for the individually developing attachment style. Taken together, features of anxiety coping, personality style and temperament may correspondent with attachment and the development of distinct psychopathologies which may over time lead to alcohol addiction.

In fact our results demonstrate a high prevalence of insecure attachment styles in alcohol addicted inpatients (IAS) compared to normative samples (e.g. [18]) with approximately two thirds of the sample having an insecure attachment style. No relevant sex differences were observed. According to the Ainsworth data which are still regarded as a standard usually approximately two thirds of a healthy sample is securely attached.

Conforming with our hypothesis and as previously described IAS subjects had significantly higher levels of trait anxiety according to the Spielberger-scale but not of state anxiety compared to alcohol dependent subjects with a secure attachment style (SAS). With regard to the correspondence of attachment style and anxiety coping we found that both IAS and SAS individuals were over-average sensitizers, continuously scanning the environment for potentially threatening stimuli in order to gain protective information. However, IAS subjects displayed a significantly higher expression of cognitive avoidance of stressful and anxiety inducing thoughts compare to SAS subjects who had average levels with regard to the normative distribution. This feature may give further evidence to the idea of alcohol addiction being an attachment disorder [31]. It may give a certain hint on a less pronounced ability to control negative emotionality related to adverse stimuli in IAS alcoholics and the function of intake of GABAergic substances such as alcohol in order to control hyper-arousal leading to addiction over time. Anxiety disorders are a frequent comorbid condition in addiction and vice versa patients with anxiety disorders or high levels of trait anxiety are more prone to substance abuse and addiction, before all alcohol and benzodiazepines [3].

A higher prevalence of personality disorders in alcohol addicted patients compared to subjects without confounding axis-I diagnosis is a well known fact. The prevalence of personality disorders was lower than estimated in our sample (15.3%), there were 8 subjects with personality disorders in the IAS group and only one SAS individual who had a paranoid personality disorder. Despite the low n of this subgroup this may be a remarkable finding of potential importance for future investigations on connections of alcoholism and personality disorders indicating the probable importance of attachment style in this respect. Yet, this should be cautiously handled as a very preliminary finding due to a low n.

Timely regarding personality as a dimensional feature, results from the correspondence of attachment style and personality styles (according to the PSI) were obviously more remarkable. We found that IAS-subjects had far over-average scores on the dimensional distribution to the pathological pole compared to the SAS group which was fairly normative on all scales (all values between percent ranks 42–59). High expression on the subscales paranoid and schizoid indicate on suppression of positive and negative emotionality, low expression of positive emotionally and a low activity of the reward-system in IAS-subjects. This appears understandable seen from an attachment related point of view. It could be hypothesized that these individuals are socially reserved because of low expectations in positive outcomes of interpersonal relationships and therefore insecure attachment may promote this style and lead to alcohol as an enhancer of a stunted reward-system.

A corresponding low reward system, a high self-centeredness and low confidence in positive outcomes of own actions and interpersonal relationships would be a characteristic of IAS subjects who scored high on dimensions related to cluster-C pathologies such as dependent, insecure, negativistic and depressive. However, these assumptions should be regarded as speculative.

All items have been rated far over-average by the IAS group and significantly lower in the SAS group. IAS alcohol addicted subjects accordingly appear to be characterized by a highly active punishment-system, a low confidence in a positive outcome of own actions and a low ability to retain or stimulate positive affectivity under adverse stimuli and frustration. There is a high sensitivity for negative evaluation by others and own needs and interests are regularly neglected. SAS individuals, however, had a significantly higher expression on the kindness / histrionic subscale, indicating a more pronounced ability of SAS subjects to show positive emotions and to rely on the ability to produce positive consequences by own actions, especially in interpersonal relationships.

This is correspondingly mirrored by the group differences revealed by the Cloninger-scale.

IAS subjects have, compared to SAS alcohol addicted inpatients, a significantly lower expression of self-directedness. This may hint on a different inner working model for interpersonal relationships in IAS individuals. The fact that IAS had a numerically higher expression of self-transcendence may indicate that coping is more related to spirituality or externalization than to faith in interpersonal relationships and their potentially beneficial effects. SAS subjects are significantly higher scorers on novelty-seeking and lower on harm-avoidance giving further insights into connections between attachment style and personality features in alcoholism. However, both groups displayed a level of co-operativeness below the average of the normative sample, showing a hampered belief in being part of a social bond. And in contrast from the PSI-findings, no difference in reward dependence could be found.

Observations of overall potentially traumatizing or adverse events in adolescence were frequently found for both groups. These findings should be interpreted cautiously as the number of observations was altogether low. One limiting factor is surely the missing specification to certain periods of adolescence which was not assessed.

Another limitation of these results is the comparatively small sample-size and that the assessment of data was mainly based on self-rating scales. However, scores and the applied openness-scale were satisfactory. Gender differences within the IAS and SAS group were not computed due to the resulting small n. Results from studies on larger samples allowing the evaluation of certain subgroups should be promising and more conclusive.

In conclusion we found in alcohol addicted inpatients, according to our initial hypothesis, a correspondence of insecure attachment styles with high trait anxiety, more dysfunctional anxiety coping and dysfunctional personality styles. Besides to comparatively high prevalence of insecure attachment styles, personality disorders were more frequently found in subjects with insecure attachment styles.

The presented results imply the potentially high importance of attachment style in the characterization of alcohol dependent men and women because of its possible high relevance for (psycho-) therapeutic strategies, individual therapeutic abilities and comorbid conditions. By separation into attachment styles significant differences in potentially dysfunctional personality styles can be observed, giving a more differentiated characterization of groups than by diagnosing personality disorders exclusively. Future research should long to prove that attachment style might be an important feature in diagnosing distinct (therapy-relevant) subgroups of alcohol addiction. Clinical routine may profit from attachment style assessments. Insecure attachment styles in alcohol dependence may contribute to poorer outcome due to dysfunctional personality styles and anxiety coping behaviour.

## Competing interests

None of the authors declares any competing interests with regard to the content of this manuscript.

## Authors’ contribution

Conception and design: DW, UH-R, BB, GH. Acquisition of data: DW, SH, Analysis and interpretation of data: DW, SH, UH-R, BB, KRE, GH. Drafting and revising of manuscript: DW, UH-R, BB, KRE, GH. Final approval: DW, BB, GH. All authors read and approved the final manuscript.
